# Targeting legume loci: A comparison of three methods for target enrichment bait design in Leguminosae phylogenomics

**DOI:** 10.1002/aps3.1036

**Published:** 2018-04-02

**Authors:** Mohammad Vatanparast, Adrian Powell, Jeff J. Doyle, Ashley N. Egan

**Affiliations:** ^1^ Department of Botany National Museum of Natural History Smithsonian Institution P.O. Box 37012, MRC 166 Washington DC 20560 USA; ^2^ Section of Plant Breeding and Genetics School of Integrated Plant Sciences Cornell University 512 Mann Library Ithaca New York 14853 USA; ^3^ Present address: Forest, Nature, and Biomass Section Department of Geosciences and Natural Resource Management Rolighedsvej 23, 1958 Frederiksberg C., University of Copenhagen Denmark; ^4^ Present address: Boyce Thompson Institute 533 Tower Road Ithaca New York 14853 USA

**Keywords:** Fabaceae, Hyb‐Seq, phylogenomics, sequence capture, target enrichment, transcriptomes

## Abstract

**Premise of the Study:**

The development of pipelines for locus discovery has spurred the use of target enrichment for plant phylogenomics. However, few studies have compared pipelines from locus discovery and bait design, through validation, to tree inference. We compared three methods within Leguminosae (Fabaceae) and present a workflow for future efforts.

**Methods:**

Using 30 transcriptomes, we compared Hyb‐Seq, MarkerMiner, and the Yang and Smith (Y&S) pipelines for locus discovery, validated 7501 baits targeting 507 loci across 25 genera via Illumina sequencing, and inferred gene and species trees via concatenation‐ and coalescent‐based methods.

**Results:**

Hyb‐Seq discovered loci with the longest mean length. MarkerMiner discovered the most conserved loci with the least flagged as paralogous. Y&S offered the most parsimony‐informative sites and putative orthologs. Target recovery averaged 93% across taxa. We optimized our targeted locus set based on a workflow designed to minimize paralog/ortholog conflation and thus present 423 loci for legume phylogenomics.

**Conclusions:**

Methods differed across criteria important for phylogenetic marker development. We recommend Hyb‐Seq as a method that may be useful for most phylogenomic projects. Our targeted locus set is a resource for future, community‐driven efforts to reconstruct the legume tree of life.

Estimating phylogenetic trees is essential for evolutionary biologists to test hypotheses and to facilitate reconstructing the tree of life. Until recently, most phylogenies were based on one to several organellar or nuclear DNA sequences (Duarte et al., 2010; Zimmer and Wen, 2015). For studies involving few taxa or a limited budget, Sanger sequencing (Sanger et al., 1977) of a few loci remains an appropriate option. However, in many cases, the backbone of phylogenetic trees may be less resolved or poorly supported, perhaps due to evolutionary processes such as recombination, hybridization, or a lack of critical phylogenetic signal at varying depths within the evolutionary history of the organisms being investigated. Therefore, many single‐ or low‐copy nuclear genes harboring phylogenetic signals useful at different phylogenetic depths are required to represent underlying evolutionary patterns and processes (Salichos and Rokas, 2013; Wickett et al., 2014; Pyron, 2015; Zimmer and Wen, 2015).

With the advancement of next‐generation sequencing technologies, obtaining genomic‐scale data is increasingly affordable. Multiple approaches have been introduced to survey genome content and collect hundreds to thousands of nuclear loci or single‐nucleotide polymorphisms for phylogenetics and population genetic studies (Egan et al., 2012; Wen et al., 2015; Heyduk et al., 2016). Target enrichment or hybridization‐based methods are some of the known “reduced representation” genome sequencing approaches (Mamanova et al., 2010; Faircloth, 2017) that have been developed and used in animals (Faircloth et al., 2012; Lemmon et al., 2012) and plants (Mandel et al., 2015; Nicholls et al., 2015; Uribe‐Convers et al., 2016) for phylogenomic studies.

In the target enrichment method, it is critical to discriminate orthologs from paralogs and to target orthologs that carry strong phylogenetic information across the study system. Multiple methods have been developed to target orthologous loci in plants for bait design and phylogenomics (Weitemier et al., 2014; Chamala et al., 2015; Folk et al., 2015; Schmickl et al., 2016). Other approaches that prioritize ortholog inference from multiple loci sourced from transcriptomes for phylogeny reconstruction (Yang and Smith, 2014) could also be useful for bait design. Recently, Kadlec et al. (2017) compared three methods for target enrichment locus selection with a focus on comparing custom versus universal marker selection approaches. Using in silico evaluations to determine the best method, they gathered empirical data across the genus *Erica* L. based on markers selected from one of three locus selection methods they investigated and ultimately showed monophyly of the genus. However, they did not produce phylogenies based on loci selected by multiple methods and were thus unable to empirically compare the relative phylogenetic signals or resolving power of loci selected by different methods.

The Leguminosae or Fabaceae is the third largest family of flowering plants after Orchidaceae and Asteraceae, comprising more than 760 genera and over 19,500 species (LPWG [Legume Phylogeny Working Group], 2017). The family is distributed in all of the world's vegetation types and is the second most prominent family after the grasses in economic value due to its legumes being used as sources of food, fodder, fuel, medicine, lumber, and soil enrichment via nitrogen fixation (Food and Agriculture Organisation, available at http://www.fao.org/pulses-2016/en/). Until recently, Leguminosae was divided into three subfamilies: Caesalpinioideae, Mimosoideae, and Papilionoideae (Lewis et al., 2005). Current research by the LPWG, an international consortium of legume systematists, revised the classification of the family to six subfamilies using the chloroplast gene *matK*: Caesalpinioideae DC., Cercidoideae Legume Phylogeny Working Group, Detarioideae Burmeist., Dialioideae Legume Phylogeny Working Group, Duparquetioideae Legume Phylogeny Working Group, and Papilionoideae DC. (LPWG, 2017). This new classification is based on the largest phylogenetic reconstruction of legume taxa to date, including approximately 20% (3696 species) of known species and over 90% of genera (698/765 genera) in the family (LPWG, 2017). The clades comprising the six subfamilies and many crown clades in the LPWG *matK* phylogeny are statistically supported (LPWG, 2017); however, the root of the family, relationships among subfamilies, and relationships among multiple internal clades remain unclear. Although the *matK* gene remains a vital tool for legume molecular systematics, the addition of multiple nuclear and chloroplast loci may clarify the evolutionary history of certain relationships.

To facilitate legume phylogenomics, we designed baits comprising hundreds of orthologous loci emphasizing the phaseoloid and millettioid legumes, a clade comprising the Phaseoleae and Millettieae tribes and the most genera‐rich subclades in the family, but one that would also have the potential for utility across all of the legumes. Our objectives were to (1) compare and contrast three popular methods for target locus selection from locus discovery to empirical phylogenetic results, (2) develop target enrichment baits for legumes, (3) validate the baits using species from several tribes within the family, and (4) introduce a workflow to facilitate similar efforts across flowering plants.

## METHODS

### Plant materials for loci selection and probe design

We used 30 transcriptomes chosen from across the legumes for target locus selection and probe design (Appendix S1). Of these, we obtained 24 transcriptomes representing 21 genera of the phaseoloid legumes, with several published already (SRP067662 in Vatanparast et al., 2016) and others in preparation (SRR5925647, 5925648, and 5925649 in Haynsen et al., unpublished data and Vatanparast et al., unpublished data). We also obtained four previously published transcriptome assemblies (Cannon et al., 2015) and two genomic coding sequences from Phytozome (https://phytozome.jgi.doe.gov/) to expand our loci discovery across the family (*Cercis canadensis* L., *Desmanthus illinoensis* (Michx.) MacMill., *Glycine max* (L.) Merr., *Glycyrrhiza lepidota* Pursh, *Lupinus angustifolius* L., and *Medicago truncatula* Gaertn.; Appendix S1). Transcriptome assembly and qualification followed Vatanparast et al. (2016).

### Locus discovery and probe design

For target loci selection, we used three pipelines available for plants: Hyb‐Seq (Weitemier et al., 2014), MarkerMiner (Chamala et al., 2015), and Yang and Smith (2014; hereafter Y&S). The Hyb‐Seq method requires transcriptomes/genome‐skimming data and a reference genome to select targeted loci (Weitemier et al., 2014). We substituted BLAST‐Like Alignment Tool (BLAT; Kent, 2002) with Pblat (available at https://github.com/icebert/pblat). We set minimum and maximum lengths of the final exons between 120 and 660 bp, respectively, removing transcripts with 90% or greater similarity to decrease the number of targeted loci with high gene copy numbers. MarkerMiner discovers putative orthologous nuclear loci based on a provided reference genome (Chamala et al., 2015). We used *M. truncatula* as the proteome reference, selecting a minimum transcript length of 700 bp, and other settings as default. The Y&S method uses a tree‐based approach to distinguish among paralogs and orthologs. To perform this method, in short, an all‐by‐all BLAST search was conducted for the 30 transcriptome assemblies with an *E*‐value of 1 followed by running Markov Cluster Algorithm (van Dongen and Abreu‐Goodger, 2012) to filter BLAST hits using an inflation value of 2 and hit‐fraction of 0.3. All clusters containing sequences from at least eight species of 30 were selected and aligned with MAFFT v7.305b using the L‐INS‐I method (Katoh and Standley, 2013). Alignments were trimmed by phyutility (Smith and Dunn, 2008), and initial phylogenetic trees were estimated by RAxML v. 8.1.2 (Stamatakis, 2014). We selected the rooted tree approach of Yang and Smith (2014), used *C. canadensis* (redbud; Cercidoideae) as the outgroup, and required a minimum of 24 species in the final trees to infer orthologs. We visualized the matrix occupancy of the orthologs to check the number of accessions per gene and selected 29 accessions (to have representatives from all species, except redbud, which is used as an outgroup) as a filter to retrieve the nucleotide sequences from orthologous trees. The final orthologous sequences were aligned using MAFFT (Katoh and Standley, 2013) and visually inspected for correct reading frame and alignment by eye. For alignments with large gaps among a subset of taxa, we also used BLAST searches to flag any gene clusters that may have incorporated multiple (alternative) splice variants that could impact bait design downstream.

To select our final loci for probe design, we checked whether any of the three methods discovered the same locus by combining the outputs of MarkerMiner, Hyb‐Seq, and Y&S into a single data set and designating unique identifications for each method in each sequence so that the original targets could be traced back to the method of inference. We used CD‐HIT‐EST (Fu et al., 2012) to retain non‐redundant sequences with over 90% similarity shared among four or more species (‐c 0.9, ‐n 4, ‐d 0, and ‐g 1). This combined set of loci was filtered to exclude clusters where original orthologs obtained from an individual method for a locus were split into different clusters by CD‐HIT‐EST. We used MAFFT to align the sequences, after which each locus was visually inspected for split orthologs.

Biotinylated RNA probes (baits) were designed by Arbor Biosciences (myBaits kit; Ann Arbor, Michigan, USA). Less than 1% of nucleotide positions were soft‐masked after subjecting sequences to RepeatMasker (Smit et al., 2013–2015) using the Leguminosae repeats database (available at http://plantrepeats.plantbiology.msu.edu/index.html). Candidate baits were aligned to the *M. truncatula* and *P. vulgaris* genomes using BLAST to remove baits that were likely to hybridize and capture to more than one genomic region. In total, 7501 120‐bp baits were designed using a tiling density of 3× and using moderate repeat‐filtration based on the BLAST results against the *P. vulgaris* genome.

### Target‐enriched genomic library preparation for probe validation

To validate our probes, we selected 25 representative species from nine tribes of subfamily Papilionoideae (Table 1). We included more species from the phaseoloid and millettioid legumes because as a clade, they represent the highest generic diversity in the Leguminosae (Lewis et al., 2005) and include a number of economically important species. Genomic DNA was extracted from silica‐dried or herbarium material using a modified cetyltrimethylammonium bromide (CTAB) method (Doyle and Doyle, 1987) or the DNeasy Plant Mini Kit (QIAGEN, Hilden, Germany). Genomic DNA was quantified with a Qubit 2.0 fluorometer (Invitrogen, Carlsbad, California, USA) using a high‐sensitivity kit and samples sonicated by QSonica (model Q800R2; Newtown, Connecticut, USA) for 45 to 60 s. The libraries were prepared using the NEBNext Ultra DNA Kit using multiplex oligos for Illumina (96 index primers; New England Biolabs, Ipswich, Massachusetts, USA) following the manufacturer's protocol. Quantification of libraries was done by Qubit, and library size validation was carried out using an Agilent 2200 TapeStation system with High Sensitivity D1000 ScreenTapes (Agilent Technologies, Santa Clara, California, USA). Libraries were hybridized with baits by multiplexing eight samples per reaction and incubated for 40 h. The KAPA HiFi HotStart Ready Mix kit (Kapa Biosystems, Wilmington, Massachusetts, USA) with the i5 and i7 primers was used for Illumina sequencing preparation. Samples were sequenced on the Illumina HiSeq 2500 platform (2 × 150 paired‐end).

**Table 1:** Sample information, sequencing summary, and target enrichment results for the Leguminosae baits set

Species	Tribe/Subtribe	Voucher (Herbarium)^a^	Collection year	Material	Raw reads (paired)	Reads mapped	% reads on target	Genes recovered (% of baits)	Final gene occupancy	No. paralogs
*Abrus pulchellus* Thwaites	Abreae	A. N. Egan 20130782 (US)	2013	Silica gel	6,895,312	2,215,590	32	479 (94.5%)	395	52
*Camptosema ellipticum* (Desv.) Burkart	Diocleae	A. Macedo 5501 (US)	1989	Herbarium	452,283	75,119	17	286 (56.4%)	222	3
*Canavalia gladiata* (Jacq.) DC.	Diocleae	A. N. Egan 12‐278 (US)	2012	Silica gel	5,662,329	1,598,816	28	494 (97.4%)	412	42
*Chadsia grevei* Drake	Millettieae	Lewis 509 (US)	1993	Herbarium	4,944,016	1,099,659	22	492 (97.0%)	409	32
*Clitoria ternatea* L.	Phaseoleae/Clitoriinae	Doyle 1600 (BH)	2010	Fresh	11,050,854	3,045,157	28	482 (95.1%)	399	34
*Cologania biloba* (Lindl.) G. Nicholson	Phaseoleae/Glycininae	Sousa 11352 (K)	1985	Herbarium	6,002,204	1,929,522	32	478 (94.3%)	395	21
*Cullen corallum* J. W. Grimes	Psoraleeae	Mitchell 7826 (PERTH)	2004	Herbarium	7,391,386	2,797,850	38	498 (98.2%)	415	41
*Deguelia negrensis* (Benth.) Taub.	Millettieae	Rimachi 11293 (US)	1995	Herbarium	6,275,432	1,986,326	32	497 (98.0%)	414	37
*Desmodium tortuosum* (Sw.) DC.	Desmodieae/Desmodiinae	A. N. Egan 11‐39 (US)	2011	Silica gel	4,179,175	1,802,826	43	477 (94.1%)	393	57
*Disynstemon paullinioides* (Baker) M. Peltier	Millettieae	Phillipson 2077 (US)	1989	Herbarium	2,591,240	1,214,746	47	474 (93.5%)	391	39
*Dolichos falciformis* E. Mey.	Phaseoleae	A. N. Egan 13‐7 (BH)	2013	Silica gel	4,698,715	1,707,082	36	496 (97.8%)	413	34
*Dunbaria punctata* (Wight & Arn.) Benth.	Phaseoleae/Cajaninae	A. N. Egan 20130731 (US)	2013	Silica gel	7,073,224	2,709,052	38	483 (95.3%)	401	56
*Glycyrrhiza glabra* L.	Galegeae	J. van der Maesen 8404 (US)	2008	Herbarium	7,982,267	2,280,024	29	503 (99.2%)	420	40
*Indigofera caudata* Dunn	Indigofereae	R. P. Clark 306 (K)	2013	Silica gel	6,135,960	1,503,920	25	496 (97.8%)	414	21
*Kennedia prostrata* R. Br.	Phaseoleae/Kennediinae	Doyle 1651 (BH)	2010	Fresh	8,949,317	3,507,185	39	490 (96.6%)	406	47
*Leptoderris micrantha* Dunn	Millettieae	van der Burgt 1576 (US)	2012	Herbarium	4,792,831	1,397,220	29	478 (94.3%)	395	47
*Lespedeza cuneata* (Dum. Cours.) G. Don	Desmodieae	Xubo 442 (CDBI)	2012	Silica gel	4,770,559	1,048,745	22	490 (96.6%)	407	37
*Macroptilium atropurpureum* (DC.) Urb.	Phaseoleae/Phaseolinae	A. N. Egan 11‐5 (US)	2011	Silica gel	11,672,490	5,637,444	48	500 (98.6%)	417	54
*Otholobium pubescens* (Poir.) J. W. Grimes	Psoraleeae	Salas 16136 (US)	1992	Herbarium	5,534,886	2,245,553	41	501 (98.8%)	418	37
*Platysepalum bambidiense* Maesen	Millettieae	Wienngien 6284 (US)	2008	Herbarium	7,985,058	1,919,772	24	499 (96.8%)	415	45
*Shuteria involucrata* (Wall.) Wight & Arn.	Phaseoleae	A. N. Egan 20130763 (US)	2013	Silica gel	4,040,622	1,499,410	37	491 (96.8%)	408	49
*Tephrosia lupinifolia* DC.	Millettieae	A. N. Egan 13‐12 (US)	2013	Silica gel	6,535,580	2,056,174	31	493 (97.2%)	410	38
*Tripodion tetraphyllum* (L.) Fourr.	Loteae	Wieringa 4847 (US)	2003	Herbarium	8,322,689	2,119,345	25	495 (97.6%)	411	24
*Vigna venulosa* Baker	Phaseoleae	Cheek 15960 (K)	2011	Herbarium	2,988,984	830,872	28	472 (93.1%)	389	45
*Wisteria frutescens* (L.) Poir.	Millettieae	K. Fetter s.n. (US)	2014	Silica gel	11,977,761	3,432,741	29	499 (98.4%)	415	70

a Herbaria are abbreviated according to Index Herbariorum (http://sweetgum.nybg.org/science/ih/).

### Assembly of targeted loci

Raw reads were quality filtered using Trimmomatic v. 0.33 (Bolger et al., 2014) with a quality cutoff of 15 in a 4‐base sliding window, discarding any reads trimmed to under 36 bp and removing adapters. Improvement in the quality of reads following trimming was assessed by FASTQC v. 0.11.5 (Andrews, 2010). We used the HybPiper pipeline (Johnson et al., 2016) to process targeted‐enrichment data using the SPAdes assembler (Bankevich et al., 2012) with a coverage cutoff of 8. In some samples with low coverage, we used a coverage cutoff of 4 with *k*‐mers 21 and 33. We checked for potential paralogs across our 25 taxa using paralog_retriever.py script provided by HybPiper. HybPiper can extract exon and flanking intron regions. We extracted exons only as well as supercontigs, which include all assembled contigs (exon and intron sequences) for each locus. Individual gene sequences (targeted loci) were aligned using MAFFT and subsequently trimmed with trimAL v1.4 (Capella‐Gutiérrez et al., 2009) to remove sequence fragments that appear in only one or a few species. Phylogenetic trees were reconstructed by using exons and supercontigs, independently, but only results of supercontigs are discussed in this article. Loci suggested as paralogous by the paralog_investigator.py script based on Exonerate results (Slater and Birney, 2005) were excluded from the main set of targeted loci.

### Species tree reconstruction

For phylogeny estimation, we used a concatenation‐, partition‐based approach using maximum likelihood (CA‐ML) and two methods, ASTRAL (Mirarab and Warnow, 2015) and singular value decomposition quartets (SVDquartets; Chifman and Kubatko, 2014), that are statistically consistent under a coalescent process. To reconstruct the CA‐ML tree, after excluding putative paralogs, we concatenated the targeted loci into a single data matrix and used PartitionFinder v. 2.0 (Lanfear et al., 2017) to find the best partitioning schemes by defining targeted loci into data blocks. The a priori data partitioning scheme for PartitionFinder was each gene as its own data partition. We used the corrected Akaike information criterion (AICc) with all branch lengths linked for model selection. We used the *rcluster* algorithm to accelerate the analysis and optimize partitioning (Lanfear et al., 2014). In total, 297 distinct data partitions with joint branch length optimization were obtained by PartitionFinder. We subsequently used RAxML v. 8.2.10 (Stamatakis, 2014) to reconstruct the CA‐ML tree and performed 500 rapid bootstraps to estimate nodal support. We also used ASTRAL III (Zhang et al., 2017), which accounts for incomplete lineage sorting (ILS) and has been shown to outperform other statistically consistent summary methods (Simmons and Gatesy, 2015; Mirarab et al., 2016). ASTRAL uses maximum quartet support for species tree estimation and calculates the local posterior probability on nodes using gene trees (Mirarab and Warnow, 2015; Sayyari and Mirarab, 2016). Gene trees for each locus were generated separately using RAxML with the GTR+GAMMA model and 200 rapid bootstraps. A species tree was obtained by ASTRAL calculating quartet scores in each node, local posterior probabilities, and number of quartet trees among the gene trees. To evaluate the performance of locus selection, we independently inferred species trees using ASTRAL on loci that originated from Hyb‐Seq, MarkerMiner, and Y&S. In addition, we used SVDquartets (Chifman and Kubatko, 2014), which has been shown to be as accurate as other species tree inference approaches (Chou et al., 2015). The concatenated data matrix was used as input for SVDquartets as implemented in PAUP* v. 4.0a build 154 (Swofford, 2002), which evaluated all possible quartets. Nodal support for the SVDquartets species tree was assessed via bootstrapping using 500 replicates in PAUP*.

Although we did not target the chloroplast genome, it is possible to obtain chloroplast loci from off‐target reads. Chloroplast loci were also assembled and recovered as above by HybPiper (Johnson et al., 2016) using the soybean chloroplast genome (GenBank accession no. DQ317523) as a reference, inclusive of 111 named loci, including tRNAs (Saski et al., 2005). For the scope of this article, we only performed unpartitioned, concatenated analysis for the plastome genes. Chloroplast loci were concatenated into a single data matrix and a tree was built using RAxML with the GTR+GAMMA model and 1000 rapid bootstraps.

Summary statistics of all data sets from bait design to target‐enrichment such as taxon occupancy, alignment length, and parsimony‐informative sites (PI) were calculated by the Alignment Manipulation And Summary (AMAS) pipeline (Borowiec, 2015). To further assess concordance and conflict among gene trees, we used the PhyParts application that estimates bipartitions across topologies (Smith et al., 2015). The concordance and conflict pie charts were made using a script from https://github.com/mossmatters/MJPythonNotebooks/blob/master/PhyParts_PieCharts.ipynb.

## RESULTS

### Comparison of the locus discovery pipelines

**Figure 1:**
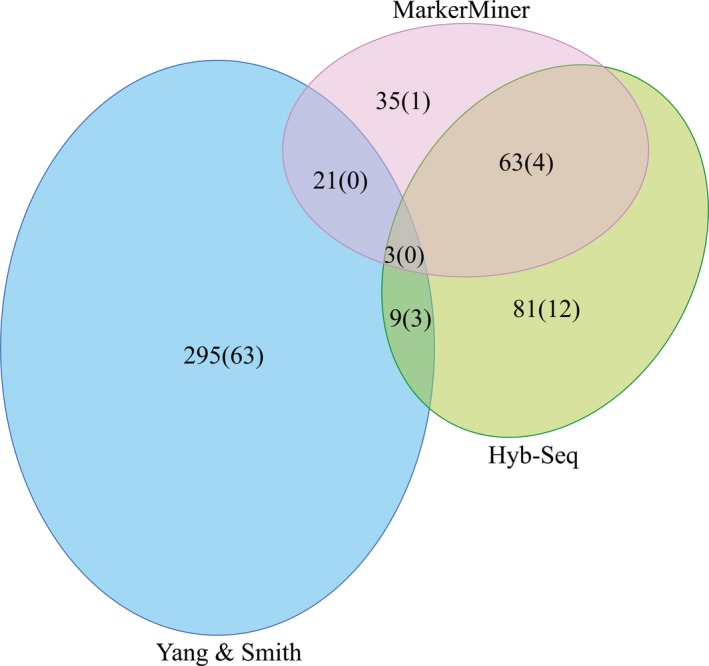
A proportional Venn diagram showing the contribution of different methods for targeted loci set development. Numbers in parentheses are the number of putatively paralogous loci from each method obtained from the recovered target enrichment loci.

**Figure 2:**
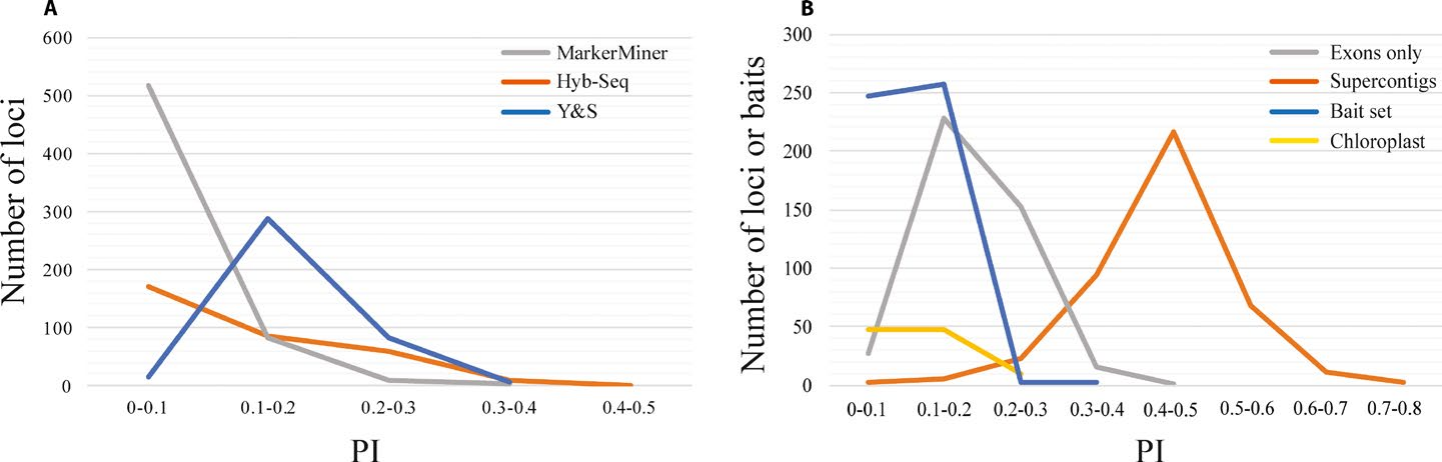
The proportion of parsimony‐informative sites for the locus discovery pipelines (A) and baits set and targeted loci (B). The *x*‐axis is the proportion of parsimony‐informative sites and the *y*‐axis is the number of sequences.

Summary statistics for each locus discovery method and data set are given in Tables 2 and 3 and Figs. 1 and 2. We retrieved 325, 612, and 390 putative single‐copy loci using the Hyb‐Seq, MarkerMiner, and Y&S methods, respectively (Table 2), in the initial gene discovery step. After clustering loci that were discovered by more than one method using CD‐HIT‐EST, we obtained 670 clusters containing four to 29 species in each cluster that averaged 1553 bp long (range: 297–3357 bp; Appendix S2). Of these, 163 clusters were excluded due to splitting of initial orthologous gene sets. Finally, we retained 507 targeted loci for probe design (Table 3, Fig. 1, Appendix S3; data available from Figshare: https://doi.org/10.6084/m9.figshare.c.4040372; Vatanparast et al., 2018). Hyb‐Seq discovered loci with the longest mean length (1816 bp), followed by MarkerMiner (1603 bp) and Y&S (1583 bp). Y&S had the highest average taxon occupancy, more than four times that of the other methods due to use of a criterion that a minimum of *n* = 24 taxa were included in the gene set. Y&S also provided the highest PI (0.17), whereas MarkerMiner had the lowest PI (0.06) among targets (Table 2). In the initial discovery phase, MarkerMiner retrieved nearly double the number of loci compared to Hyb‐Seq and Y&S. However, in the final set of targeted loci, Y&S contributed the highest number of putative orthologs (*n* = 328; 84% forwarded), followed by Hyb‐Seq (*n* = 156; 48%) and MarkerMiner (*n* = 122; 19%) (Table 2, Fig. 1).

**Table 2:** Summary statistics for the three loci discovery methods.^a^

Target discovery method	No. of target regions^b^	Mean no. of taxa per loci	Mean alignment length (bp)	Proportion of parsimony‐informative sites	No. of putative paralogs in targeted loci^c^
Hyb‐Seq	325 (156)	6	**1816**	0.10	15 (9.6%)
MarkerMiner	612 (122)	6	1603	0.06	**5 (4.1%)**
Yang & Smith	**390 (328)**	**26**	1583	**0.17**	66 (20%)

a Results in bold represent the optimal value among comparisons.

b Numbers in parentheses are the number of regions included in the final baits design.

c Percentages in parentheses are percent of paralogs discovered within the target regions included in the final baits design.

**Table 3:** Summary of target enrichment results

Comparative unit	No. of target regions	Mean no. of taxa per locus	Mean alignment length for exons/supercontigs (bp)	Proportion of parsimony‐informative sites for exons/supercontigs
Targeted loci for bait design	507	15	1406	0.09
Targeted loci recovered	506	24	1717/2542	0.19/0.43
Putative orthologs	423	23	1743/2607	0.18/0.43
Plastome genes (off‐targets)	104	22	810	0.11

### Target enrichment

Of 25 species used in this study, DNA was extracted from fresh leaf material for two species, from silica‐dried material for 11 species, and from herbarium specimens for 12 species. A herbarium specimen of *Camptosema ellipticum* (Desv.) Burkart, collected in 1989 and deposited at the Smithsonian Institution herbarium (US), had the lowest DNA quality before library preparation, possibly due to DNA degradation, incomplete grinding, or the presence of secondary compounds. After hybridization and sequencing, *C. ellipticum* had the lowest number of raw reads and the lowest number of recovered targeted loci (44% missing; Table 1). To test the effect of the missing data of *C. ellipticum* on analyses, we re‐analyzed the CA‐ML and ASTRAL species tree reconstructions, both with and without *C. ellipticum*; tree topologies were identical (data not shown) and therefore we retained *C. ellipticum* in subsequent analyses.

**Figure 3:**
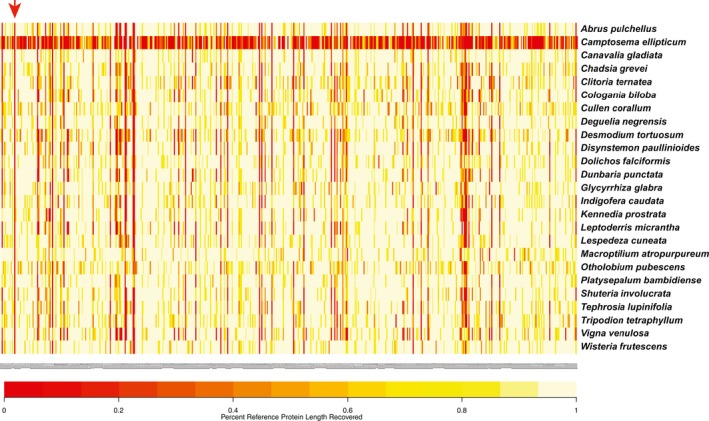
Heat map showing target enrichment efficiency of 507 loci for 25 species from nine tribes of the subfamily Papilionoideae recovered by HybPiper using the Burrows–Wheeler Alignment method. Each column is a locus and each row is a taxon, with species names presented to the right. The color spectrum shows the length of sequence recovered by the pipeline normalized (divided by) the length of the reference (target) gene, from red (0, no gene recovery) to light yellow (1, full recovery). Locus 314 had zero recoveries in all species, shown by the red arrow.

The results of the target enrichment are summarized in Table 1. Using the reference assembly approach of HybPiper, we recovered 506 of 507 targeted loci across 25 species, with only *C. ellipticum* exhibiting considerable locus loss (286 loci, 56.4% of baits captured; Fig. 3, Appendix S4). For all other taxa, we recovered at least 93% of targeted loci, with *Glycyrrhiza glabra* L. (503 loci, 99.2%) being the highest (average 94.94%, mode 94.3%, median 96.8%; Table 1). Only one locus (number 314), which was retrieved by Y&S in the locus discovery step, was not recovered in any species (Fig. 3). Of 506 loci, 281 (55%) were recovered in all 25 species reported here, followed by 165 recovered in 24 taxa (32%) (Table 1, Appendix S5).

The mean number of taxa for which sequences were obtained (taxon occupancy) for each of the 506 recovered targeted loci was 24, with a mean alignment length of 1717 bp and proportion of PI of 0.19 for exons only, more than double that of the baits alone (PI = 0.09; Table 3). Extracting supercontigs increased the mean alignment length to 2542 bp and PI to 0.43 (Table 3, Fig. 2). Within 506 recovered loci, 83 loci were flagged as putatively paralogous by HybPiper, ranging from three in *C. ellipticum* to 70 in *Wisteria frutescens* (L.) Poir. (Table 1, Appendices S6 and S7). Of the 83 putative paralogous genes, the Y&S method produced the highest number of putative paralogs (*n* = 66; 20%) and MarkerMiner the least (*n* = 5; 4.1%) (Table 2, Fig. 1). The final data set of putative orthologs included 423 loci with an average alignment length of 2607 bp and a PI of 0.43 (Table 3).

### Off‐target chloroplast gene assembly

We recovered sequences from 104 chloroplast loci from off‐target reads with a mean taxon occupancy of 22 taxa, a mean alignment length of 810 bp (range: 69–10,346 bp), and a PI of 0.11 (Table 3, Appendix S8). The majority of the 104 chloroplast loci recovered were coding regions without introns, but some comprised tRNAs and intergenic spacers, and a few included intronic sequence. In the case of *ycf1* and *ycf2* genes, we excluded very long insertions from *Disynstemon paullinioides* (Baker) M. Peltier before phylogenetic analysis. We could not recover any chloroplast sequences for *Cologania biloba* (Lindl.) G. Nicholson. The total alignment length of the concatenated chloroplast matrix was 84,340 bp.

### Species tree reconstructions

**Figure 4:**
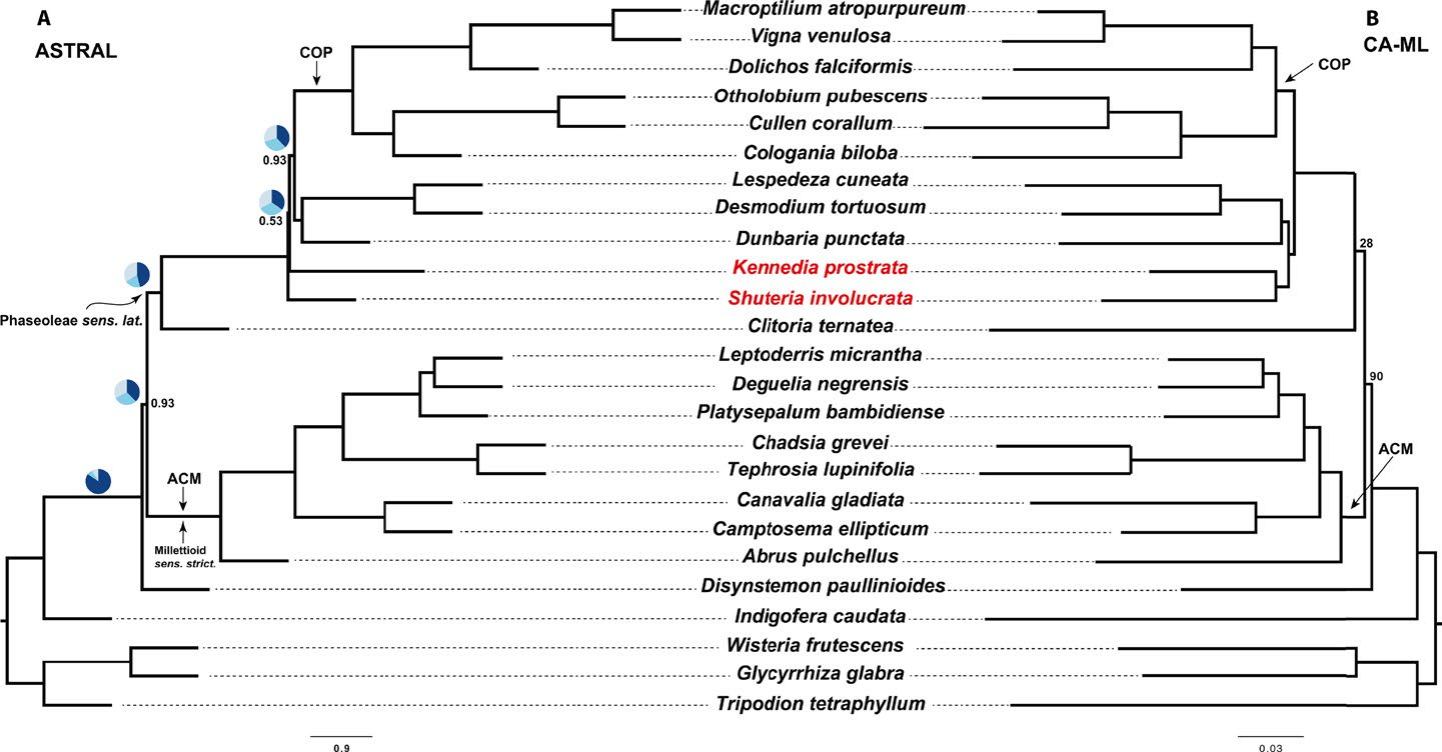
Species trees based on the ASTRAL (A) and CA‐ML (B) methods. The ASTRAL tree is based on 423 nuclear loci, and CA‐ML is a concatenation of 423 nuclear loci from 25 species. Numbers are local posterior support (ASTRAL) or bootstrap (CA‐ML). Branches with no numbers have local posterior support of 1.0 or bootstrap support of 100, respectively. Pie charts (in the ASTRAL tree) show relative frequencies of the three quartet topologies around the branch in gene trees for selective nodes. Species in red show incongruency between the ASTRAL and CA‐ML trees. COP:* Cologania biloba*,* Cullen corallum*,* Dolichos falciformis*,* Macroptilium atropurpureum*,* Otholobium pubescens*, and *Vigna venulosa*. ACM:* Abrus pulchellus*,* Camptosema ellipticum*,* Canavalia gladiata*,* Chadsia grevei*,* Deguelia negrensis*,* Leptoderris micrantha*,* Platysepalum bambidiense*, and *Tephrosia lupinifolia*.

**Figure 5:**
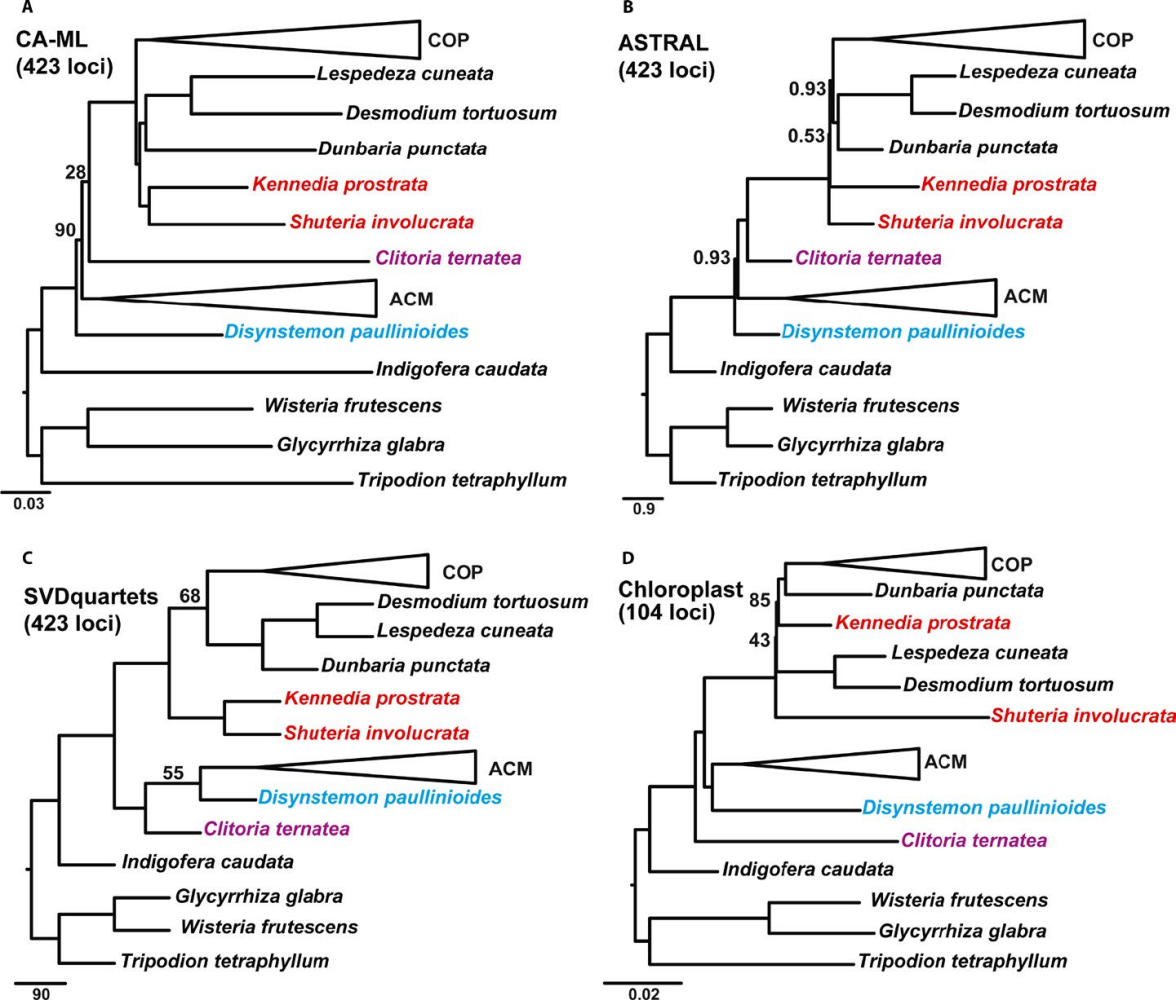
Species tree based on 423 nuclear loci (A–C) and 104 chloroplast loci (D). (A) CA‐ML. (B) ASTRAL. (C) SVDquartets. Nodes without numbers have local posterior support of 1.0 or bootstrap support of 100; other levels of support are shown along the branches. Collapsed triangles (COP and ACM) are as in Fig. 4. The place of incongruency among the trees is shown in red, blue, and purple names. (D) COP does not include *Cologania biloba*, as this taxon is missing from the chloroplast data set. Relationships within the collapsed nodes are identical in all trees.

**Figure 6:**
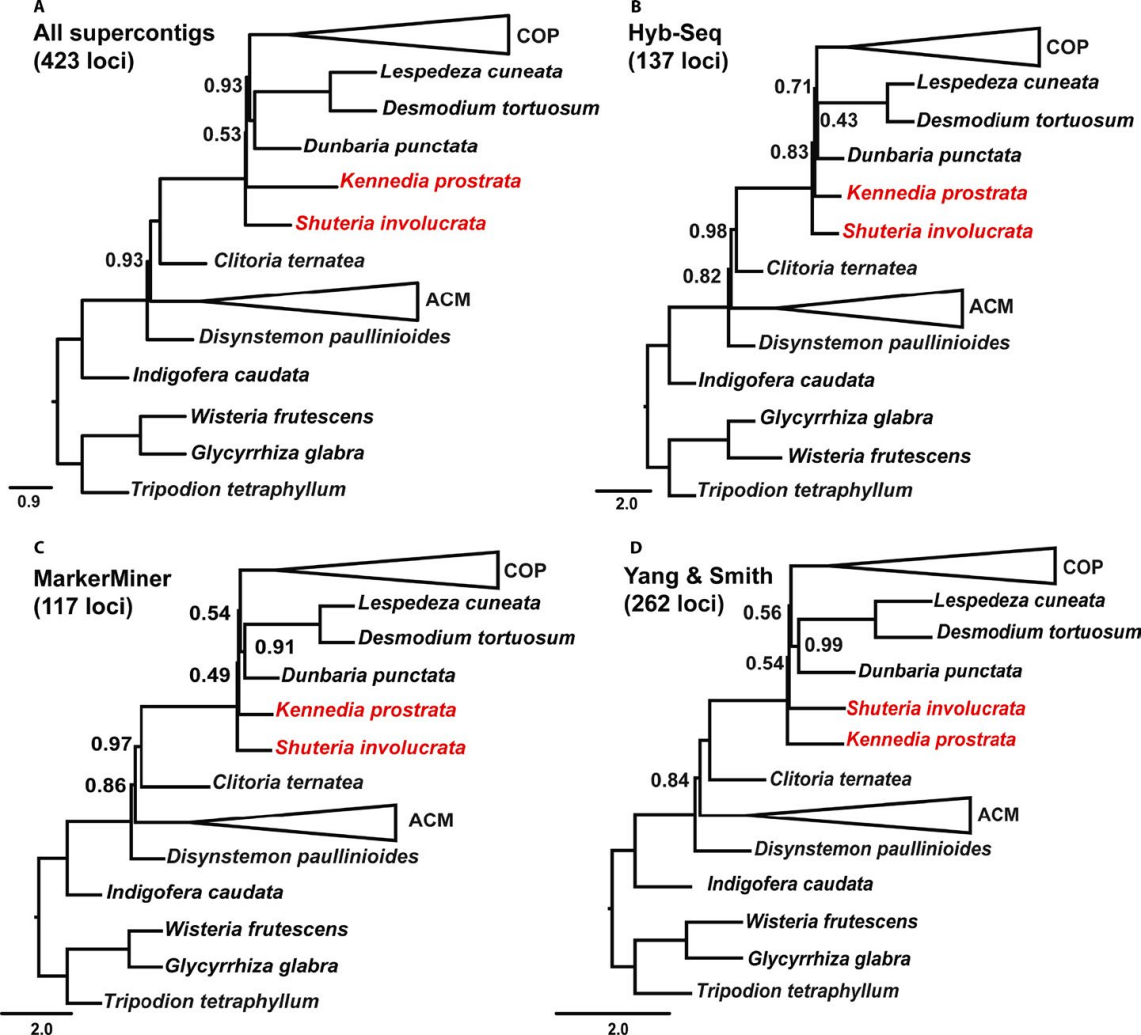
ASTRAL species tree based on supercontigs (exons plus introns) of 423 nuclear loci (A), Hyb‐Seq (137 loci, B), MarkerMiner (117 loci, C), and Yang & Smith (262 loci, D). Nodes without numbers have local posterior support of 1.0; other levels of support are shown along the branches. The place of incongruency among the trees is shown in red. Collapsed triangles (COP and ACM) are as in Fig. 4 and relationships within the collapsed nodes are identical in all trees.

**Figure 7:**
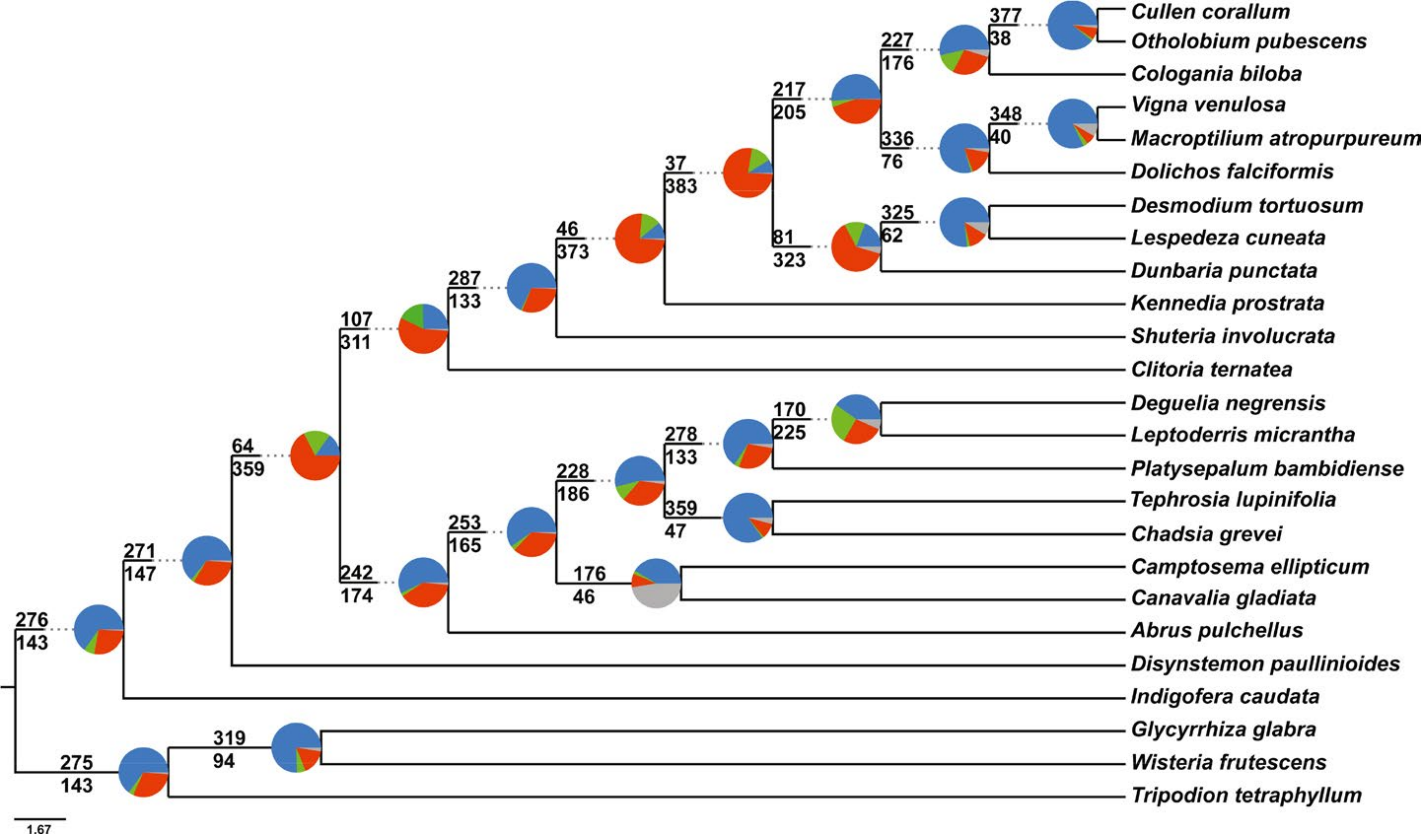
ASTRAL species tree based on 423 gene trees with a summary of conflicting and concordant homologs. For each branch, the number of homologs concordant (top) or in conflict (bottom) with the species tree at each node is indicated. Pie charts at each node present the proportion of homolog support for that clade (blue), the main alternative (green), the remaining alternatives (red), and the proportion that inform (conflict or support) this clade that have less than 50% bootstrap support (gray).

The CA‐ML method yielded an alignment length of 1,102,958 bp from 423 loci with 487,554 PI (44.2%) for supercontigs in comparison to the 737,309 bp with 134,679 PI (18.26%) for exons only. Of the 423 gene trees (data available from Figshare: https://doi.org/10.6084/m9.figshare.c.4040372; Vatanparast et al., 2018), 205 gene trees were missing at least one taxon (see gene occupancy, Table 1; Appendix S4). Two clades were strongly and robustly supported across all phylogenetic analyses: (1) the core Phaseoleae sensu Schrire (Lewis et al., 2005; designated herein as COP) comprised subtribe Phaseolinae (sensu Lackey, 1981), represented by *Vigna* Savi, *Macroptilium* (Benth.) Urb., and *Dolichos* L., as sister to a clade comprising tribe Psoraleeae (*Otholobium* + *Cullen*) and *Cologania* Kunth; and (2) the *Abrus* Adans. *+* Millettieae clade (designated herein as ACM) comprised tribes Abreae (*Abrus*), Diocleae (*Canavalia* Adans. and *Camptosema* Hook. & Arn.), and several members of Milletteae sensu stricto (s.s.) (Fig. 4). Topologies inferred from the CA‐ML, ASTRAL, and SVDquartets analyses of 423 loci and from the chloroplast data set were largely similar with some discordance along internal nodes that represented key relationships among previously circumscribed tribes (Fig. 5). In the CA‐ML tree, *Shuteria involucrata* (Wall.) Wight & Arn. is sister to *Kennedia prostrata* R. Br., a clade that is then sister to a clade comprising *Dunbaria punctata* (Wight & Arn.) Benth. + Desmodieae (*Desmodium tortuosum* (Sw.) DC. and *Lespedeza cuneata* (Dum. Cours.) G. Don) (Fig. 4B). In the ASTRAL tree, *Dunbaria* + Desmodieae are sister to the core Phaseoleae (COP), with *Shuteria* Wight & Arn. and *Kennedia* Vent. as early‐diverging lineages with weak local posterior support and very short branch lengths (Fig. 4A). In the SVDquartets tree, *Shuteria* is strongly supported as sister to *Kennedia*, like the CA‐ML tree, but sister to a monophyletic clade in which COP is sister to *Dunbaria* + Desmodieae (Fig. 5C). SVDquartets strongly supports *Clitoria ternatea* L. as sister to *Disynstemon paullinioides*, a clade that is sister to the millettioids (ACM). In contrast, the CA‐ML analysis of the 104 chloroplast loci resolves *Disynstemon* R. Vig. as sister to ACM, a clade that is then sister to the phaseoloid legumes, with *Clitoria* L. resolved as sister to a millettioids and phaseoloids clade (Fig. 5D), whereas the CA‐ML and ASTRAL trees support *Disynstemon* as sister to the remaining millettioids and phaseoloids, with *Clitoria* nested within (Fig. 5A, B). Comparisons of the phylogenetic trees based on loci discovered by the three methods (Hyb‐Seq, MarkerMiner, and Y&S) are presented in Fig. 6B–D. The main topologies from each of the three methods are almost identical (Fig. 6B–D), with the only difference being the swapping of *Kennedia* and *Shuteria*, which differs in the Y&S tree (Fig. 6D) in comparison to the other methods and “overall” phylogenetic tree (Fig. 6A). Furthermore, support for the clade of *Dunbaria* sister to the Desmodieae is much reduced in Hyb‐Seq (local posterior of 0.43) relative to the other methods (MarkerMiner: local posterior of 0.91; Y&S: local posterior of 0.99; 423‐loci tree: local posterior of 1.0; Fig. 6). Analysis of concordance and conflicts suggests that a discordant phylogenetic signal exists across loci mapped over the species tree (Fig. 7).

## DISCUSSION

Choosing appropriate loci for target enrichment is no trivial task, especially given the impact of evolutionary phenomena such as whole genome or single‐gene duplication, hybridization, differential gene birth and death, or recombination, all of which can confound phylogenetic signal through ortholog/paralog conflation. Fortunately, the effects of some of these phenomena on species tree inference can be mitigated during marker selection and development. Here, we compare and contrast three popular methods for locus selection, paying attention to the varying strengths and weaknesses of these methods and making efforts to trace paralogs and orthologs from the reference set through marker validation. We compared 30 transcriptomes for initial locus discovery, empirically validated our loci using 25 legume taxa from across papilionoid legumes emphasizing phaseoloid and millettioid clades, and ultimately present a set of 423 putatively orthologous nuclear genes for phylogenomic use across the legume family.

### Efficiency of marker development methods

Target enrichment sequencing relies upon the selection of a set of putatively orthologous genes whose sequences capture both the conservative (single copy or orthologous) and variable (phylogenetically informative) nature of the genome, thus enabling phylogenetic resolution across a desired evolutionary breadth. Early efforts of marker selection mostly centered on utilizing highly conserved loci designed for broad taxonomic utility (Faircloth et al., 2012; Lemmon and Lemmon, 2013). With the development of open source marker selection pipelines, designing custom probe sets for phylogenomics, in particular at lower taxonomic levels, is becoming more accessible (Faircloth, 2017). Recently, several methods have been developed to aid in locus discovery, each with their relative strengths and weaknesses. Choice of method should be selected based on project objectives (Kadlec et al., 2017). In this study, we assessed the Hyb‐Seq, MarkerMiner, and Y&S pipelines using transcriptomes sampled across the legumes.

Hyb‐Seq was one of the first methods to “skim” genomic and transcriptomic data for locus discovery (Weitemier et al., 2014). It is easy to implement and can be used for locus discovery and probe design of nuclear and organellar genomes. In our study, Hyb‐Seq targets had the longest alignment length (Table 2) and medium PI; similar results were found in the comparison of Hyb‐Seq and MarkerMiner by Kadlec et al. (2017). Hyb‐Seq's flexibility to designate target length allows it to retrieve flanking introns beyond exons and may be one reason why Hyb‐Seq produced the longest mean target length.

MarkerMiner (Chamala et al., 2015) is a modular and user‐friendly pipeline that can be run locally or via web or docker applications (available at https://github.com/vivekkrish/markerminer-webapp). Currently, MarkerMiner's utility is limited by the selection of a few reference plant genomes. According to our results, MarkerMiner targets were the least phylogenetically informative (and thereby most conservative) among the three methods (Fig. 2), a finding corroborated by Kadlec et al. (2017) and likely due to the fact that MarkerMiner uses single‐copy, “core orthologs” deemed highly conserved across angiosperms as a reference set (De Smet et al., 2013; Chamala et al., 2015). MarkerMiner also produced the fewest paralogs (five loci; 4.1%) as detected by HybPiper, again likely due to the conserved, single‐copy nature of the reference set.

The Y&S (2014) method was originally designed for phylogenomic analysis of gene clusters from transcriptomic or genomic data, not as a locus discovery tool in and of itself. However, the fact that Y&S does not use a reference genome and can make use of a full spectrum of input data, coupled with its phylogeny‐based orthology assessment, makes it an appealing method for locus discovery. Like Hyb‐Seq and MarkerMiner, the Y&S method uses similarity‐based clustering to initially define orthologous clusters (via all‐by‐all BLAST). The Y&S method had the most discovered loci pass through our initial optimization filters and the highest taxon occupancy relative to other methods (although this is a direct result of criteria for marker selection in our workflow). It also had the highest PI, but the shortest mean alignment length, just behind MarkerMiner (Table 2). On the other hand, among 83 paralogous loci detected by HybPiper in our targeted loci, Y&S had the most genes (20%) flagged as paralogs, even after our optimization step. This may be due to the inclusion of separated “inclades” representing orthologous, but duplicated, gene alignments as separate loci in the target loci set. Finally, Y&S requires considerably more analytical resources and time compared to MarkerMiner and Hyb‐Seq.

Comparing the phylogenetic trees of these three methods (Fig. 6B–D), MarkerMiner had the most comparable results to the “overlap” phylogenetic tree based on 423 loci (Fig. 6A), whereas Hyb‐Seq lacked strong nodal support for the *Dunbaria* + Desmodieae clade found in all other analyses. The Y&S tree resolved a different topology with respect to *Kennedia* and *Shuteria* in comparison to the others. However, the branch lengths resolving these relationships are extremely short in all trees, and the nodal support for both MarkerMiner and Y&S is extremely low, suggesting that this node may actually be a hard polytomy. Alternatively, the short branch lengths and poor nodal support may suggest an ancient hybridization or polyploidization followed by differential gene loss across loci.

Taken together, the similar species trees assessed from the individual methods suggest that all three programs selected are useful for developing loci for phylogenomic inference. However, methods differed in the number of loci that satisfied our criteria, with Y&S having the most and MarkerMiner the least (Table 2). Furthermore, each method differed in the proportion of phylogenetically informative sites, the mean alignment length, and the number of putative paralogs (Table 2). If tallying the best in each of several criteria (see Table 2), Y&S would be the obvious winner. However, the intense computational load required for this method can make it prohibitive, and although Y&S leads in the number of mean taxa per locus, this metric is inflated due to required criteria during locus discovery. In addition, Y&S had the most putative paralogs flagged. Although each of the three methods would likely yield successful loci for target enrichment, we suggest that Hyb‐Seq may be a good choice for most phylogenomic projects; its minimal computational load returned the longest average length of discovered loci and intermediate levels of PI and putative paralogs coupled with a relatively high number of loci passing optimization. By examining inferred species trees based on loci discovered from all three methods, we extend the comparisons of Kadlec et al. (2017) and present a complete analysis of empirical gene trees from nuclear targets as well as off‐target chloroplast loci. Kadlec et al.'s (2017) AllMarkers/BestMarkers method was not included in our comparisons, as this method was published just after we completed bait design, sequencing, and analysis.

### Efficiency of bait design and targeted loci

Reduced representation methods for phylogenomics all suffer from locus dropout to varying degrees; however, exon capture has been shown to suffer far less than other methods (Harvey et al., 2016). Of our 507 targeted loci, only one locus was not captured in all our accessions (Fig. 3). Furthermore, locus recovery was over 93% for all accessions but one (*Camptosema*), a result likely due to library quality rather than bait inefficiency. Interestingly, *Glycyrrhiza*, one of our outgroup taxa, had the highest locus recovery (99.2%) of all accessions, with the other two outgroup taxa, *Wisteria* Nutt. (98.4%) and *Tripodion* Medik. (97.6%), on the high end as well (Table 1, Appendix S4).

In addition to the inclusion of breadth at the locus discovery phase, we produced full transcriptome assemblies of all 30 taxa used for locus selection, rather than the two to four that many studies use. In a comparison of exome DNA sequencing technologies, Clark et al. (2011) showed that the method with the highest level of probe tiling (NimbleGen; Roche Sequencing Solutions, Madison, Wisconsin, USA) increased enrichment efficiency and sensitivity to small variants. Similarly, we believe that by tiling probes (we tiled 3×) and allowing site variants to be represented in tiled probes, the probability of capturing a wider taxonomic breadth increased. Our high locus recovery across Papilionoideae suggests that our bait sets may be useful family‐wide (Lavin et al., 2005). By using pipelines such as HybPiper (Johnson et al., 2016), it is possible to harvest at least partial introns and intergenic regions that may be more informative than the exons alone (Fig. 2). For example, by including introns, overall resolution and nodal support are increased in our phylogenetic trees; with the exons‐only data set, the ASTRAL tree had five nodes lacking complete support, whereas including introns decreased that number to three nodes (Fig. 4, Appendix S9). Moreover, by including introns in the final data set, the target enrichment approach may be more useful at the species level, with the expectation of more variation offered by intronic regions; in our case, including intronic regions more than doubled the PI (44.2% vs. 18.26%; Fig. 2B).

### Gene tree discordance and paralogs

As an evolutionary unit, selective pressures differ across genes within an organism as well as across lineages and over time. As a result, gene trees can differ from each other and from the species tree. Multiple processes such as gene or genome duplications, ILS, hybridization, and chromosomal rearrangements can interfere with accurate inference of the species tree (Smith et al., 2015; Springer and Gatesy, 2015). The recent development of multi‐species coalescent methods in phylogenomics take into account some of the issues stated above, such as ILS; however, conflicts between species and gene trees are still common, even at the phylogenomic scale (Gatesy and Springer, 2014).

The results of our concordance‐conflict analysis show that in multiple nodes, only a minority of the gene trees are concordant (Fig. 7), supporting the suggested topology, even though bootstraps and local posterior support for those nodes are high (e.g., nodes involving *Kennedia prostrata* and *Shuteria involucrata*). In ASTRAL, this discordance can also be investigated by the branch length and the frequency of the total number of quartets in all gene trees that support the main topology, the first alternative, and the second alternative in each node, presented by pie charts in selective nodes in our ASTRAL tree (Fig. 4A). Even though local posterior support of the ASTRAL tree for some nodes is relatively high (e.g., 0.93 for the node of Phaseoleae sensu lato [s.l.] and millettioid s.s.), the frequency of quartets are nearly equal (Fig. 4A). Those nodes that are characterized by high gene tree discordance, as illustrated in nearly equal quartet frequencies, also tend to have short branches in the ASTRAL tree (Fig. 4A); as such, very shallow branch lengths are a direct indicator of the discordance among the gene trees in ASTRAL, of which even a custom Yule prior model (lambda) did not improve the situation (data not shown). The concordance‐conflict analysis corroborates this finding (Fig. 7). Statistical binning could be helpful by reducing gene tree estimation error when gene trees have reduced bootstrap support (Mirarab et al., 2014), and clustering genes based on their functions could be another way of improving gene tree estimation (Mandel et al., 2015).

### Toward Leguminosae phylogenomics: examples from phaseoloid and millettioid legumes

The latest and largest phylogeny of the Leguminosae based on the chloroplast *matK* gene suggested a new subfamily‐level classification, expanding the number of subfamilies from three to six, but relationships among subfamilies and among multiple clades are still unresolved (LPWG, 2017). These unclear relationships could be due, in part, to several independent whole genome duplication events that happened in the early stages of Leguminosae evolution (Cannon et al., 2015), rapid radiation of lineages in the Paleogene period (Lavin et al., 2005), or low variation of the single chloroplast gene, which is assumed to represent the maternal line for the majority of sampled taxa (LPWG, 2017). By capturing numerous nuclear loci at the genome‐scale and at the same sampling scale as presented by LPWG (2017), we may be able to represent the full evolutionary history of how Leguminosae evolved into the diverse, species‐rich family it is today (LPWG, 2013).

A few phylogenomic studies have recently been completed in legumes such as *Acacia* Mill. (Williams et al., 2016), *Inga* Mill. (Nicholls et al., 2015), *Medicago* L. (de Sousa et al., 2014), and *Oxytropis* DC. (Shavvon et al., 2017). However, no extensive locus set has yet been published or validated beyond the genus level in legumes. In this pilot study, we introduce more than 500 nuclear loci designed from species across the family and validated using 25 species mainly selected from the diverse millettioids and phaseoloids within the subfamily Papilionoideae (Table 1). Although we do not address the unresolved relationships among the six subfamilies discussed above, we illustrate utility of the target enrichment approach among the Phaseoleae s.l. clade, or “phaseoloid” legumes, one of the largest of the 28 recognized tribes in Papilionoideae. Phaseoleae comprises over 120 genera and more than 2200 species, including important crops such as soybean, common bean, and winged bean (Lewis et al., 2005). Phylogenetic relationships among phaseoloids, Millettieae s.s., and basal millettioids have historically been difficult to resolve (Lewis et al., 2005; Stefanović et al., 2009; Egan et al., 2016). The phylogenetic trees based on our targeted loci resolved several core groups (presented as COP and ACM clades in this study), which are in line with previous studies; however, conflicts still exist among the species tree reconstruction methods and data sets (Fig. 5). For example, CA‐ML (Fig. 5A) and SVDquartets (Fig. 5C) support *Shuteria* as sister to *Kennedia*, a finding also discovered by Li et al. (2013), whereas ASTRAL (Fig. 5B) and the concatenated chloroplast loci (Fig. 5D) placed *Shuteria* as sister to remaining Phaseoleae + Desmodieae, reminiscent of relationships determined by Doyle and Doyle (1993). Moreover, the position of *Clitoria* and *Disynstemon* are different in our ASTRAL, CA‐ML, SVDquartets, and chloroplast trees (Fig. 5). Some of these topologies, like those recovered by SVDquartets and chloroplast loci, are consistent with previous studies (de Queiroz et al., 2015; LPWG, 2017). However, CA‐ML (Fig. 5A) and ASTRAL (Fig. 5B) support *Disynstemon*, a monospecific genus endemic to Madagascar, as sister to remaining millettioids + phaseoloids, a result supported by a combined analysis of nuclear ribosomal ITS and morphological characters (Schrire et al., 2009). These discordances are reflected in the near‐equal quartet frequencies and shallow branch length of nodes surrounding *Disynstemon* in the ASTRAL tree (Fig. 4A) as well as in the concordance‐conflict analysis (Fig. 7), which could be due to any combination of missing data (gene/species), orthology/paralogy conflation, ILS, whole genome duplication, recombination, rapid radiation, and/or ancient hybridization resulting in “chloroplast capture” (Egan and Crandall, 2008; Galtier and Daubin, 2008; Doyle and Egan, 2010; Li et al., 2013). Some loci may be less useful as phylogenomic markers due to their inclusion in large gene families that diversify readily through recombination and differential gene birth and death processes that scramble phylogenetic signal. As an example, a brief glimpse into the function of some of our loci flagged as paralogs found several heat shock proteins (data not shown). Although programs like ASTRAL account for ILS (Mirarab and Warnow, 2015) and we excluded putative paralogs identified by HybPiper, determining the cause of these discordances can be difficult. Additional taxon sampling may help to resolve shallow nodes and reconstruct robust relationships among this important and diverse group, work that is currently in progress.

## CONCLUSIONS

We compared three methods for targeted loci selection using representative species from the largest subfamily of legumes. We selected target loci using transcriptomes, targeting hundreds of nuclear loci and validated them across nine tribes and 25 species of subfamily Papilionoideae. Our results suggest that all methods can be useful for locus discovery, but that perhaps Hyb‐Seq has advantages with respect to ease of use, computational load, locus length, and moderate levels of parsimony‐informative sites coupled with lower numbers of paralogous loci. Our phylogenetic trees are consistent with previous studies, validating the utility of the target enrichment approach for legume phylogenetics. Nevertheless, careful attention is needed in reference to orthology/paralogy conflation, gene/species tree discordance, and making efforts to account for common phenomena in plants such as hybridization and polyploidization. That being said, the use of many genes for phylogenetic study moves inference of evolutionary history from the gene tree to the species tree and enables a more holistic view of evolutionary history. Our bait set is publicly available for purchase through Arbor Biosciences. We encourage its use across various phylogenetic studies, efforts that could benefit the legume community toward implementing phylogenomic approaches at various taxonomic levels that would enable integration across markers from multiple studies to achieve a higher understanding of legume evolution and reconstruct a community‐level Leguminosae tree of life. Such international collaborations and knowledge sharing across the legume community are highly beneficial, as exemplified by LPWG (2013, 2017).

## DATA ACCESSIBILITY

Data, including baits alignments and gene trees, are available from Figshare (https://doi.org/10.6084/m9.figshare.c.4040372). All scripts and workflow of the current study are available from GitHub (https://github.com/Smithsonian/Fabaceae_Phylogenomics_workflow).
