# Supplementary figures and images for: Targeting legume loci: A comparison of three methods for target enrichment bait design in Leguminosae phylogenomics
Source: Appl Plant Sci. 2018 Apr 2;6(3):e1036. doi: 10.1002/aps3.1036 (PMC5895186; doi:10.1002/aps3.1036)

Appendix S2. Number of clusters before baits design and their alignment length distribution.

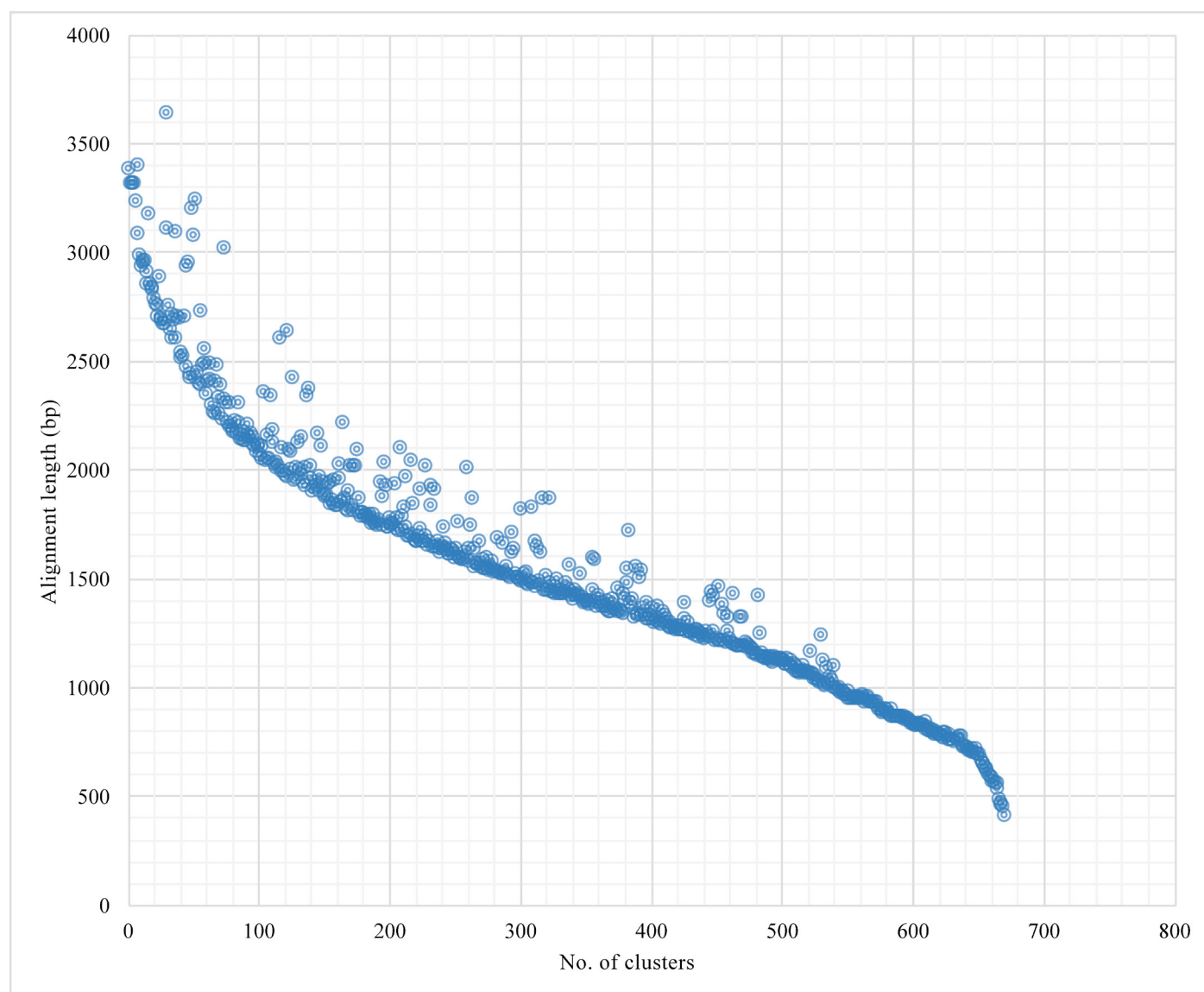

Supplement: Supplementary file 2 [file APS3-6-e1036-s002.pdf]

Appendix S5. Recovered genes per species base. Only gene 314 is missing for all 25 species (Fig. 3).

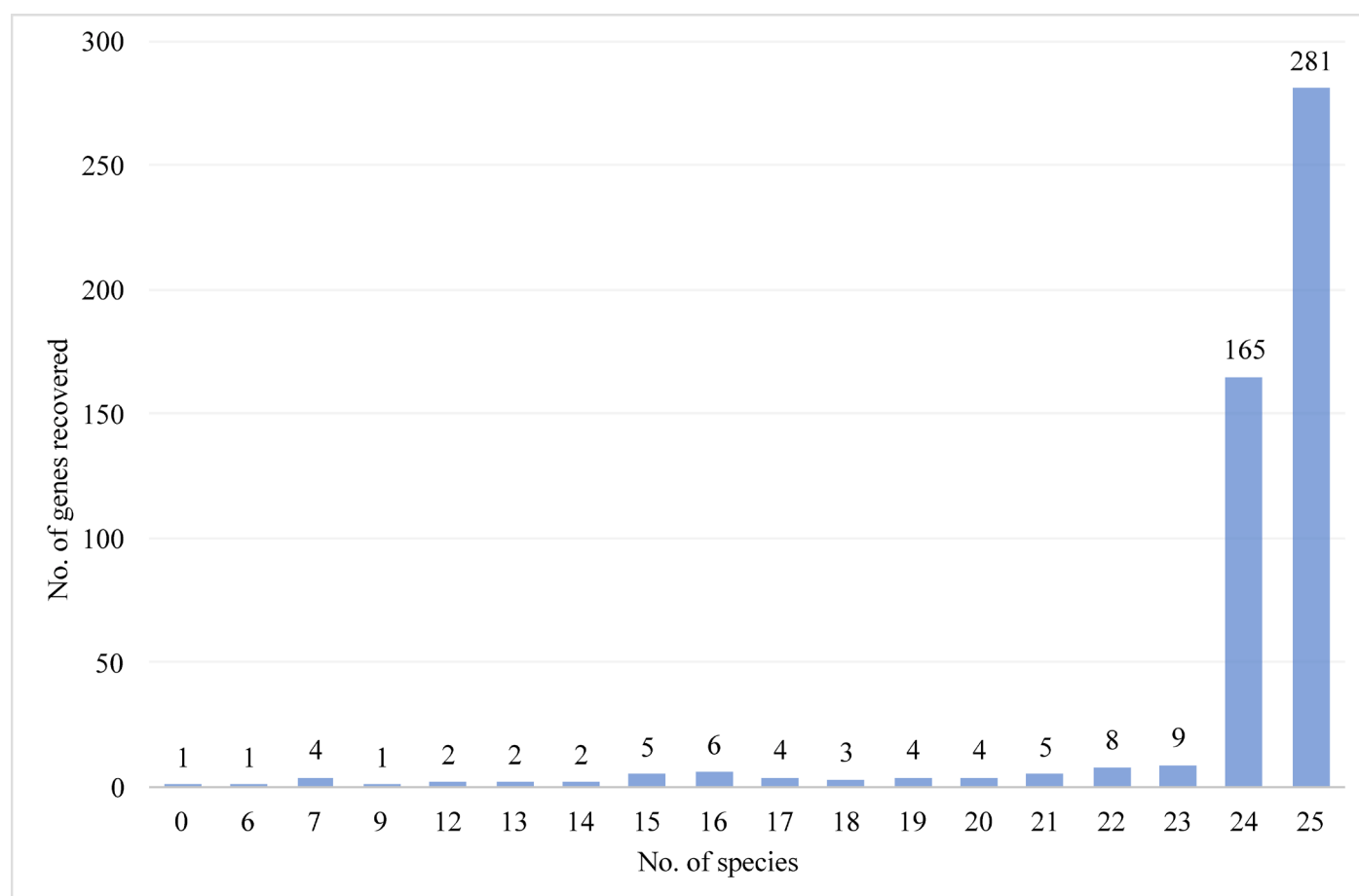

Supplement: Supplementary file 5 [file APS3-6-e1036-s005.pdf]

Appendix S6. Number of paralogs per species.

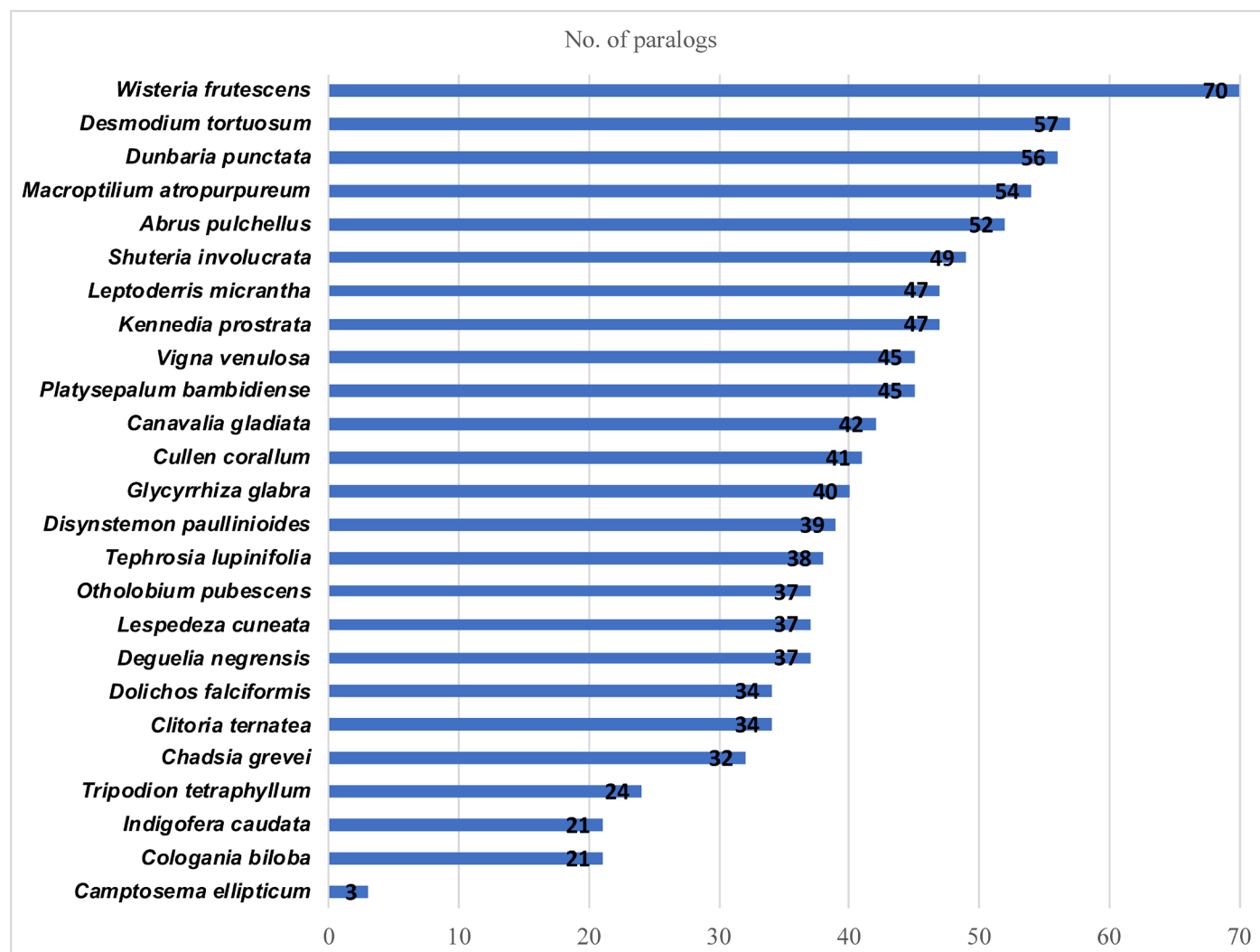

Supplement: Supplementary file 6 [file APS3-6-e1036-s006.pdf]

Appendix S7. Number of paralogs per gene.

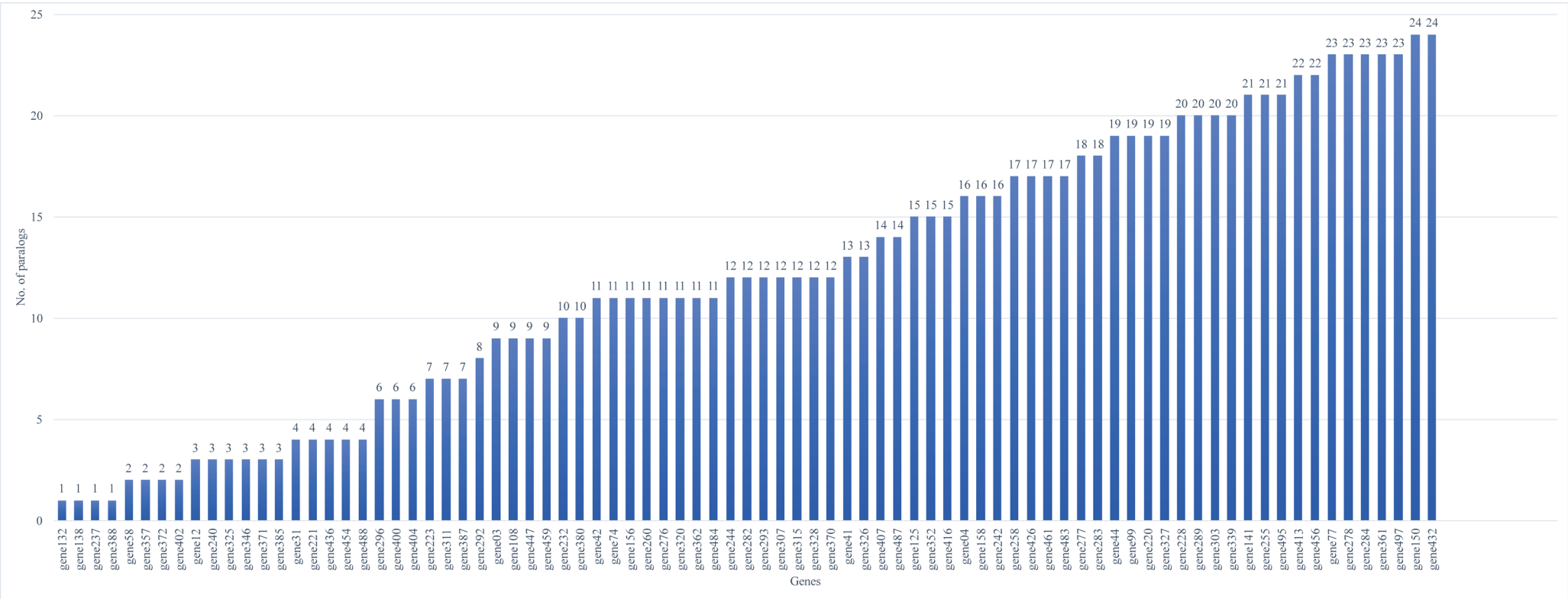

Supplement: Supplementary file 7 [file APS3-6-e1036-s007.pdf]
