# Supplementary material for: Targeting legume loci: A comparison of three methods for target enrichment bait design in Leguminosae phylogenomics
Source: Appl Plant Sci. 2018 Apr 2;6(3):e1036. doi: 10.1002/aps3.1036 (PMC5895186; doi:10.1002/aps3.1036)

Appendix S9. ASTRAL (A) and CA-ML (B) species tree using exons-only sequences of 423 nuclear loci. For details, see the legend of Fig. 4.

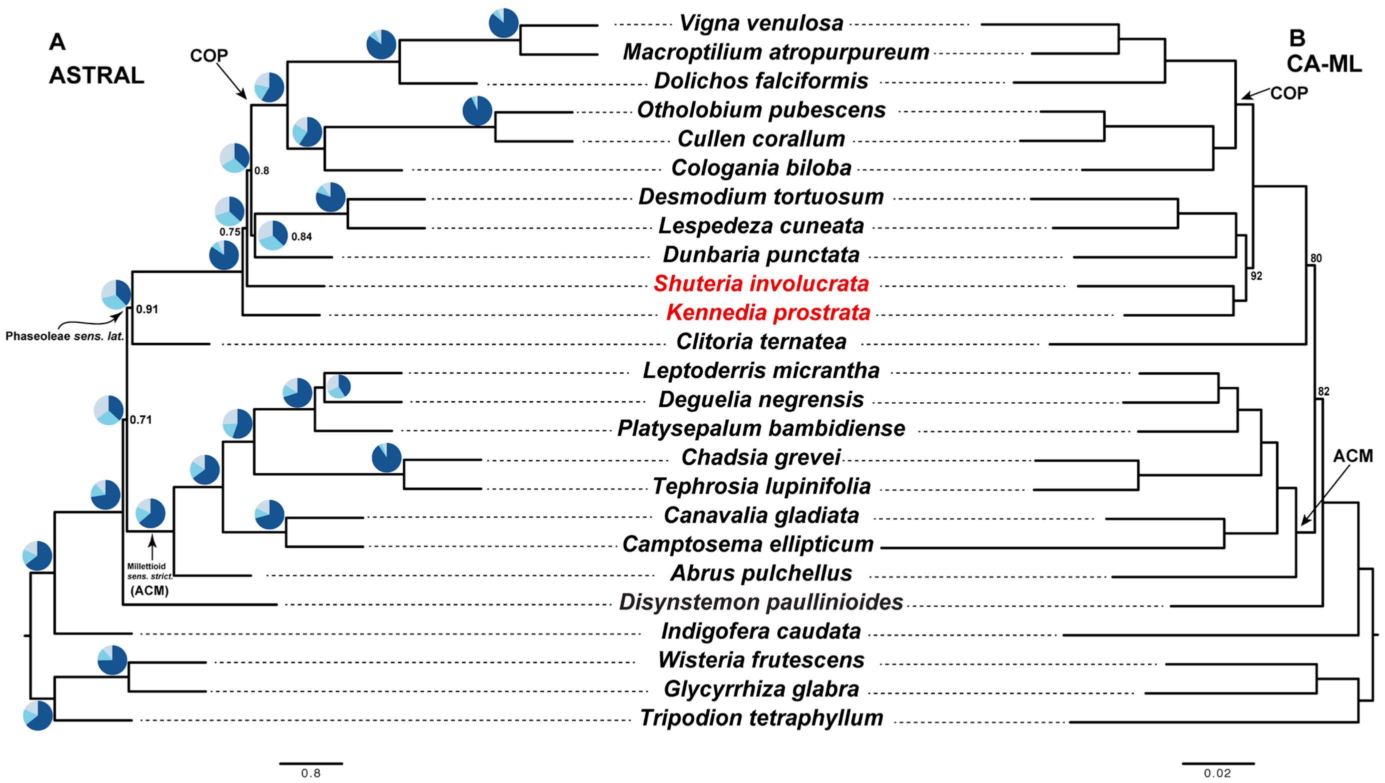

Supplement: Supplementary file 9 [file APS3-6-e1036-s009.pdf]
